# Supplementary material for: Dynamic control of high-voltage actuator arrays by light-pattern projection on photoconductive switches
Source: Microsyst Nanoeng. 2023 May 16;9:59. doi: 10.1038/s41378-023-00528-w (PMC10185468; doi:10.1038/s41378-023-00528-w)
Supplement: Supplementary file 6 — Supplementary information [file 41378_2023_528_MOESM6_ESM.pdf]

SUPPORTING INFORMATION

Dynamic control of high-voltage actuator arrays by light-pattern projection on photoconductive switches

Vesna Bacheva[1,2], Amir Firouzeh[3], Edouard Leroy[3], Aiste Balciunaite[2], Diana Davila[2], Israel Gabay[1], Federico Paratore[2,4], Moran Bercovici[1,*], Herbert Shea[3,*], Govind Kaigala[2,5,*]

[1]Faculty of Mechanical Engineering, Technion – Israel Institute of Technology, 3200003 Haifa, Israel

[2]IBM Research Europe - Zurich, Säumerstrasse 4, 8803 Rüschlikon, Switzerland

[3]Soft Transducers Laboratory (LMTS), Ecole Polytechnique Fédérale de Lausanne (EPFL), 2000 Neuchâtel, Switzerland

[4]Current affiliation: Laboratory of Soft Materials and Interfaces, ETH Zürich, 8093 Zürich, Switzerland

[5]Current affiliation: University of British Columbia, Vancouver, Canada

[*]Corresponding authors

Contents

S1. Characterization of ZnO

S2. Time response of photoactuated FEEO gate electrodes

S3. Photoactuated HAXEL array

S4. Captions for movies

S5. References

S1. Characterization of ZnO

In this work, we used hydrogenated amorphous silicon (a-Si:H) as a photoconductor material. However, we conducted some of our initial tests also using zinc oxide (ZnO). Specifically, we measured the ratio of light to dark conductance as a function of the illumination wavelength and compared it with that of a-Si:H, as shown in Fig. S1. While a-Si:H responds to light in the entire visible spectrum, ZnO responds significantly stronger to UV-light (e.g. a 1000-fold more than to red light) due to its energy band structure[1]. This may be advantageous in applications where decoupling between actuation and imaging is desired (e.g., actuation in UV with fluorescence imaging in the red).

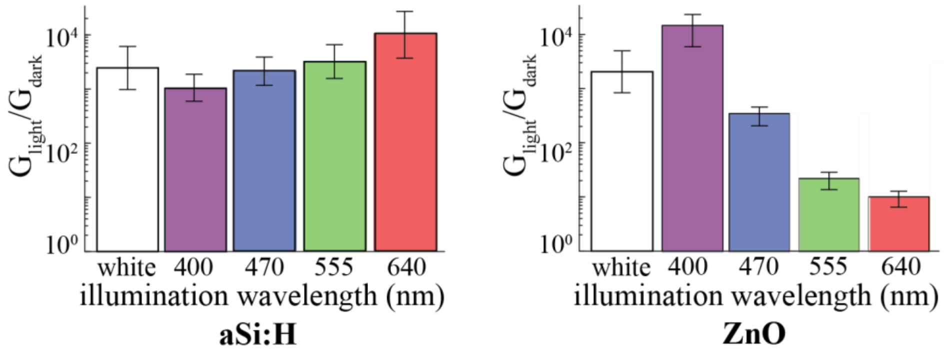

Fig. S1. Comparison of the the light to dark conductance ratio as a function of illumination wavelength for a-Si:H and ZnO photoconductive switches. The power density of the light was measured using optical power meter and is: 73.1 mW/cm^2 for white, 17.2 mW/cm^2 for red, 23.3 mW/cm^2 for green, 18.1 mW/cm^2 for cyan, 16.5 mW/cm^2 for violet, and the presented conductance ratio is normalized by the light intensity at each wavelength. Both switches had a 100 µm gap and were subjected to 200 V. We subjected each switch to three cycles of 10s in the dark, followed by 10s upon illumination, and based on the obtained results, we calculated an average dark and light conductance. The error bars represent the 95% confidence interval of the mean (with at least 10 switches, and 3 on/off cycles in each).

S2. Time response of photoactuated FEEO gate electrodes

As described in the paper, the gate electrode together with the switch can be regarded as an RC circuit for their electrical response. The switch should operate such that in the absence of illumination, due to the low conductance in the switch, the resulting RC time is higher than the operating AC time, resulting in a low voltage drop across the capacitor and thus negligible EOF velocity. In contrast, during the ON-state, the conductivity of the switch is sufficiently high such that the RC time is much shorter than the operating AC time, allowing most of the voltage to drop across the capacitor and induce EOF.

We here estimate the RC time of a gate electrode connected to a photoconductive switch. Fig. S2 shows a schematic of the system that can be modeled as an RC circuit[2]. The capacitor C is composed of the capacitance of the electric double layer (C_{EDL}) in series with the dielectric capacitance C_d. The resistor R is composed of the resistance of the liquid channel ($R_{channel}$) in series with the resistance of the switch (R_{switch}), which decreases when exposed to light. We calculate the values of C_{EDL} using the relation[2]

$$C_{EDL}=\frac{\varepsilon_l A}{\lambda_{EDL}},$$

where ε_l is the dielectric constant of the liquid, A is the area of the gate electrode (200 µm in diameter), and λ_{EDL} is the thickness of the electric double layer. For our buffer (10 mM acetic acid and 1 mM NaOH), the λ_{EDL} is approximately 10 nm.[3] Similarly, we calculate the dielectric capacitance C_d

$$C_d=\frac{\varepsilon_d A}{d},$$

where ε_d is the dielectric constant of the thin SiON layer, and d (600 nm) is its thickness. Using these relations, we estimate that the total capacitance of the system is approximately 10 pF. We measured the resistances in the system and obtained values of 150 MΩ, 15 GΩ, and 10 MΩ for the channel's resistance, the switch's dark resistance, and the switch's light resistance, respectively. Using these values for C and R, we obtain that the resulting RC time of our system in the dark is approximately 150 ms, and upon illumination is approximately 1.6 ms.

The voltage drop over the capacitor subjected to an AC voltage $V_s(t) = V_s\cos(\omega t)$ is given by[4]:

$$V_c(t) = V_s \frac{1}{\sqrt{1 + (RC\omega)^2}} \sin(\omega t + \varphi),$$

where ω is the operating frequency, and φ is a phase constant ($\tan(\varphi) = 1/RC\omega$). The time-average voltage drop over the capacitor is given by:

$$V_{c,avg} = \omega \int_0^{1/\omega} V_c(t)dt.$$

Considering that we use an AC voltage with a 25 Hz operating frequency (i.e., 40 ms period), the average voltage drop over the capacitor is only 12 % of V_s in the absence of illumination, and 92 % of V_s when the switch is illuminated. This allows the switch to be effective in AC, and is consistent with the observations in our experiments.

A single gate electrode can be also connected to several switches as shown in Fig. S3. Each switch is connected to a different power supply. By illuminating the desired switch while keeping the others dark, it is possible to select the operating voltage for the gate electrodes, and thus the EOF velocity.

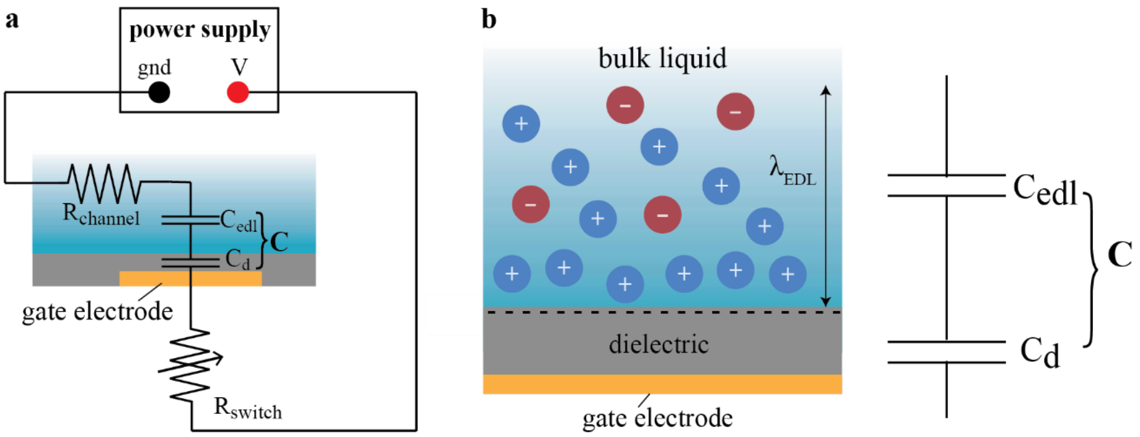

Fig. S2. RC model of the gate electrode in series with a photoconductive switch. The capacitor C is composed of the capacitance of the electric double layer (C_{EDL}) in series with the dialectic capacitance C_d. The resistor R is composed of the resistance of the liquid channel ($R_{channel}$) in series with the resistance of the switch (R_{switch}). **b** Schematic of the electric double layer on top of a gate electrode, and their equivalent capacitance.

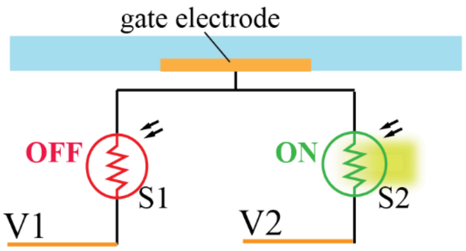

Fig. S3. Schematic illustration of a single gate electrode connected to two power supply via two switches S1 and S2. By illuminating the desired switch, the gate electrode is supplied with the corresponding voltage. We show two power supplies for simplicity, but this concept is extendable to multiple supplies.

S3. Photoactuated HAXEL array

Fig. S4a shows a 3D rendering of the different layers of a 5x5 HAXEL array. Fig. S4b shows a photograph of a fabricated HAXEL array, and the LED matrix used for its control. Fig. S5 shows the array of photoconductive switches that are located under the array.

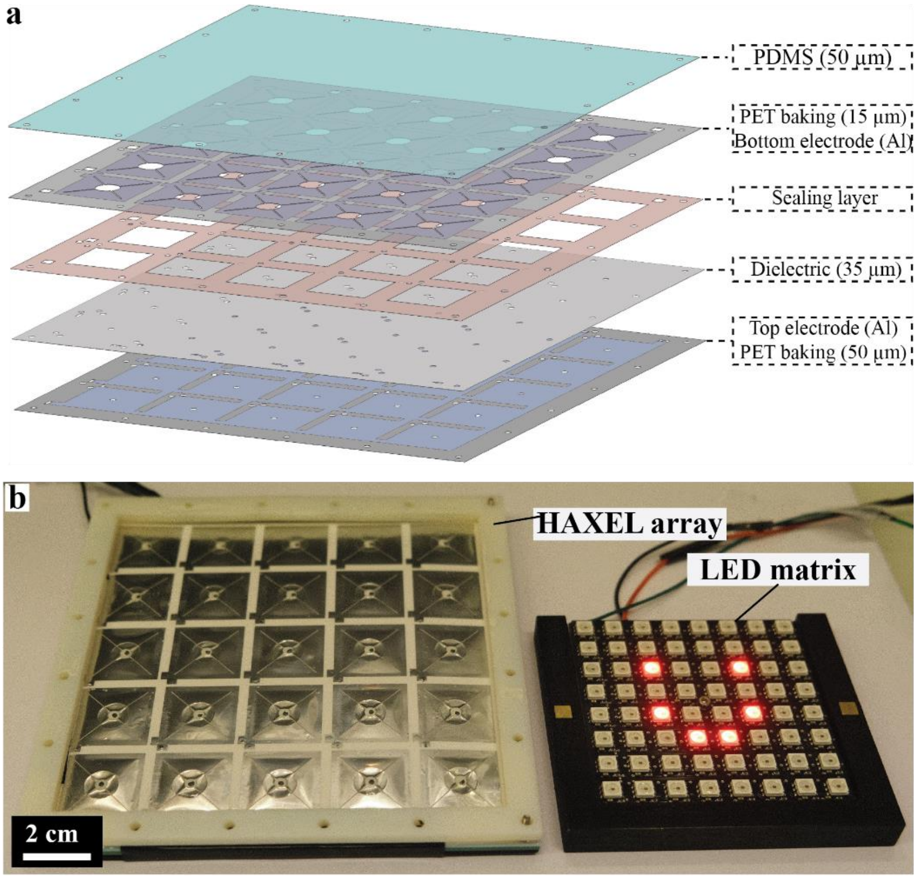

Fig. S4 a Exploded view of a 5x5 HAXEL array that is composed of five main layers. **b** Photograph of the HAXEL array and the LED matrix, which normally it is fixed under the array using magnets.

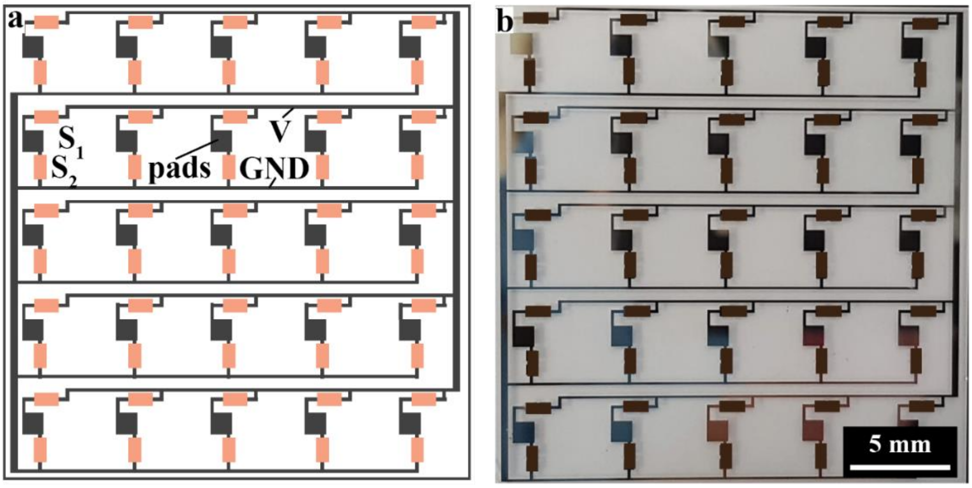

Fig. S5. a Schematics of the layout of the photoconductive switch used for the HAXEL array. Each actuator is connected to two switches S1 and S2. All S_1 switches are connected to the power line (voltage V), and all switches S_2 are connected to a common ground. Metallic pads are used to interface with the actuator array. **b** Photograph of microfabricated photoconductive switch array.

S4. Captions for movies

Movie S1. Individual addressing of gate electrodes. A single square-shaped gate electrode gives rise to an electroosmotic dipole flow[2,5]. The yellow square indicates which electrode is turned ON by illumining its corresponding switch. Each frame of the video was background subtracted and obtained by the superposition of 5 frames of the raw data time-lapse.

Movie S2. Photoactuated flow patterns with one power supply. Flow patterns obtained by actuating several gate electrodes simultaneously. Each frame of the video was background subtracted and obtained by the superposition of 5 frames of the raw data time-lapse.

Movie S3. Photoactuated flow patterns with two power supplies. Flow patterns generated by several photoactuated gate electrodes connected to two power supplies generating flow upward (red square) or downward (blue square). By using two switches per electrode, we can select which electrode is ON and the direction of the flow on each electrode. Each frame of the video was background subtracted and obtained by the superposition of 5 frames of the raw data time-lapse.

Movie S4. Photoactuated HAXEL array. Deformations obtained by a photoactuated 5x5 HAXEL array. The inset shows the light patterns of S1 switches only, and indicates which actuator is ON. For better visualization, we digitally masked the regions between the actuators with a black grid.

Movie S5. Motion of a ball on a programmable topography. The trajectory of the ball is dictated by the topography of an elastic membrane, which is suspended on top of a 5x5 HAXELs array. To move the ball form one point to another, we turn ON the actuator at its origin, and turn OFF the actuator at its desired location. The inset shows the light patterns corresponding to S1 switches only.

S4. References

1. Park, S., Kim, B. J., Kang, S. J. & Cho, N.-K. Photocurrent Characteristics of Zinc-Oxide Films Prepared by Using Sputtering and Spin-Coating Methods. *J. Korean Phys. Soc.* **73**, 1351–1355 (2018).
2. Paratore, F., Bacheva, V., Kaigala, G. V. & Bercovici, M. Dynamic microscale flow patterning using electrical modulation of zeta potential. *Proc. Natl. Acad. Sci.* **116**, 10258–10263 (2019).
3. Khademi, M. & Barz, D. P. J. Structure of the Electrical Double Layer Revisited: Electrode Capacitance in Aqueous Solutions. *Langmuir* **36**, 4250–4260 (2020).
4. Rawlins, J. C. CHAPTER 7 - RC Circuit Analysis. in *Basic AC Circuits (Second Edition)* (ed. Rawlins, J. C.) 217–256 (Newnes, 2000). doi:10.1016/B978-075067173-6/50008-0.
5. Boyko, E., Rubin, S., Gat, A. D. & Bercovici, M. Flow patterning in Hele-Shaw configurations using non-uniform electro-osmotic slip. *Phys. Fluids* **27**, 102001 (2015).
